# Ecological Factors Influencing Decision-Making Among Middle-Aged and Older Adults: A Neurocognitive Approach

**DOI:** 10.7759/cureus.82598

**Published:** 2025-04-19

**Authors:** Muddsar Hameed, Nahin Sani, Arsalan Sharif, Muhammad Allahyar Malik, Haniya Ihsan, Kasim Syed Jafri, Fatima Saqib Rashid, Musa Khan Bungish, Ashar Sami, Talal Ehsan, Muhammad Shahreyar, Syeda Masooma Naqvi, Marium Nadeem Khan

**Affiliations:** 1 Neuroscience, Brain Tech Clinic and Research Center, Islamabad, PAK; 2 Medical School, Shifa International Hospital, Islamabad, PAK; 3 General Practice, Tbilisi State Medical University, Tbilisi, GEO; 4 Medical School, Shifa College of Medicine, Islamabad, PAK; 5 Internal Medicine, CMH Lahore Medical College, Lahore, PAK; 6 Internal Medicine, CMH Kharian Medical College, Kharian, PAK; 7 Internal Medicine, Great Western Hospital NHS Foundation Trust, Swindon, GBR; 8 Clinical Psychology, Shifa Tameer e Millat University, Islamabad, PAK

**Keywords:** decision-making capacity, ecological factors, neurocognitive approach, older adults, psychological well-being

## Abstract

Introduction: Decision-making capacity (DMC) significantly influences the autonomy and well-being of middle-aged and older adults. While extensive research has addressed cognitive determinants of decision-making, less attention has been given to ecological factors, especially within non-Western contexts. This study aims to assess ecological influences (social environment, emotional states, stress levels) on decision-making competence among the older population in Pakistan, employing a neurocognitive approach to address gaps in culturally diverse research.

Methods: A quantitative cross-sectional study was conducted from September to October 2024, involving 100 adults aged ≥45 years from Rawalpindi and Islamabad. Participants completed a structured questionnaire incorporating demographic information, the Adult Decision-Making Competence (A-DMC) scale, the Satisfaction with Life Scale (SWLS), the Geriatric Depression Scale (GDS), and assessments of ecological and cognitive factors. Data was analyzed using IBM SPSS Version 26 (IBM Corp., Armonk, NY), employing descriptive statistics, correlations, independent sample t-tests, ANOVA, and regression analyses.

Results: Marital status and living arrangements significantly influenced social environment perceptions (p = 0.03), stress levels (p = 0.05), and cognitive engagement (p = 0.01). Married individuals reported better social environments and lower stress than single participants. Individuals living with family showed higher subjective well-being (M=19.61±4.53) compared to those living alone (M=17.33±7.09) or with friends (M=13.20±6.69; F=4.65, p<0.01). Age groups showed non-significant trends in decision-making scores, with seniors (65+ years) scoring lower (M=33.75±20.33) compared to pre-seniors (M=45.21±14.05) and middle-aged adults (M=44.35±13.33; p=0.06). Regression analysis indicated minimal predictive value of social environment, ecological context, and physical environment for decision-making competence (R²=0.014, p=0.711).

Conclusion: The findings underscore the significant roles of marital status and living contexts in shaping psychological well-being, social interactions, and stress in middle-aged and older adults. Decision-making appears less directly influenced by ecological contexts, indicating potential resilience due to life experience. Future research should employ longitudinal designs and broader samples to enhance understanding and generalizability.

## Introduction

The term "decision-making capacity" (DMC) describes a person's capability to comprehend and retain information pertinent to a particular decision and use that information to communicate and make a decision. This skill is essential throughout life, especially for middle-aged and older adults, as they face complex decisions impacting their autonomy, quality of life, and well-being [1, 2]. As people age, they face more impactful decisions about health, finances, and relationships, often with limited recovery from poor outcomes. Increased life expectancy, a focus on independence, and dispersed family networks heighten the need for sustained decision-making competence in older adults [3]. Tens of thousands of people in the United States are faced with situations where they need to make critical medical decisions on their own but lack the support of family members or surrogates. This is because they are cognitively impaired, either acutely or chronically, and are unable to make decisions for themselves [4]. These people make up 3% of residents in nursing homes, 16% of patients in intensive care units, and an unknown number of people in various settings who must make end-of-life decisions [4]. However, when DMC is compromised, it can lead to a loss of autonomy, as others may need to make decisions on behalf of the older adult. This loss of control can be particularly distressing and may negatively impact an individual's self-worth and dignity [5]. Additionally, techniques to evaluate DMC in older adults are becoming increasingly necessary due to the ageing population trends and associated prevalence of chronic diseases that may affect decision-making [6].

Global ageing trends and increased life expectancy are significant challenges in older adults' decision-making capacity, particularly regarding independent living [7]. Age-related cognitive changes generally lead to declining decision-making capacity among older adults, potentially compromising their ability to make sound judgments about independent living [8].

Diminished cognition, particularly in executive functions such as working memory, processing speed, and attention, has been consistently linked to impaired decision-making abilities in older adults [9]. Recent studies have demonstrated that these cognitive changes negatively impact older adults' performance on various decision-making tasks, including the Iowa Gambling Task, financial decision-making, and real-world decision-making scenarios [10]. The evidence suggests that age-related cognitive declines make it more challenging for older adults to process complex information, evaluate multiple options, and make advantageous choices, especially in time-pressured or complex situations. Furthermore, these cognitive changes can increase older adults' susceptibility to framing effects, potentially making them more vulnerable to how information is presented [11]. However, despite age-related cognitive declines, many older adults maintain or even improve their decision-making capacity for independent living due to accumulated life experience, emotional regulation, and compensatory strategies. For example, research indicates that older adults often maintain decision-making competence despite cognitive declines [12]. Demonstrated that technology-based decision support tools help older adults effectively navigate complex healthcare and financial decisions. Additionally, it was revealed that certain aspects of decision-making competence, such as resistance to sunk costs, can improve with age, suggesting that life experience mitigates cognitive decline [13]. These findings collectively highlight the valuable skills older adults possess in decision-making contexts.

The concept of ecological rationality challenges traditional views on ageing and decision-making capacity (DMC) by emphasizing the crucial role of environmental factors [14]. This perspective argues that simple decision strategies can be effective in many natural environments, potentially mitigating the effects of age-related cognitive changes. However, there is a notable gap in the literature regarding using ecological momentary assessments (EMAs) to capture real-time, context-specific data on decision-making processes in older populations, particularly in non-Western settings. Furthermore, research has shown that various ecological factors, such as living conditions and exposure to green spaces, significantly influence cognitive function and DMC in older adults [15,16]. In addition, recent studies suggest that the impact of cognitive decline on decision quality in older adults is not straightforward but depends on the fit between decision strategies and specific environmental contexts. Key findings indicate that strong social support networks, community living environments, regular healthcare access, and higher socioeconomic status contribute to preserving DMC. For instance, a study by RAND Europe highlights the effectiveness of community-based support systems (CBSIs) in promoting healthy ageing, emphasizing the benefits of ageing in place and social engagement in middle-income countries [17]. Similarly, a WHO report discusses various care models for older adults, including institutional care. While it generally advocates for community-based solutions, it also acknowledges that, in some cases, institutional settings can provide specialized care and continuous monitoring that may benefit specific individuals with complex needs [18]. As populations globally continue to age, resolving this balance is critical to informing policies and interventions that support DMC in older adults.

Furthermore, recent research indicates that cumulative stress exposure (CSE) significantly affects cognitive functioning in older adults. Higher CSE is associated with poorer baseline cognitive performance and slower global cognition and executive function decline over time, particularly among females [19]. These findings highlight the importance of considering emotional states and stress levels when assessing DMC in older populations.

Most DMC research has been conducted in Western contexts, leaving a significant gap in understanding how cultural and socioeconomic factors influence decision-making in countries like Pakistan. This study addresses this gap by assessing how ecological factors, including social environment, emotional state, stress levels, and cognitive abilities, influence decision-making capacity in older adults in Pakistan using a neurocognitive approach. This study aims to contribute to a more culturally diverse understanding of DMC, enhancing the global applicability of existing theories and models.

Objectives

To assess the influence of ecological factors (e.g., social environment, physical environment, emotional state, and stress) on decision-making capacity among middle-aged and older adults in Pakistan.

To examine the relationships among cognitive abilities, subjective well-being, depression, stress, and decision-making capacity across different demographic groups (age, gender, marital status, and employment status).

To investigate the role of social context (living alone, with family, or friends) in shaping older adults' decision-making capacity and psychological well-being.

Rationale

Decision-making capacity (DMC) is critical to older adults' independence and well-being. Global demographic shifts toward ageing populations have heightened the importance of understanding factors influencing decision-making among older adults. Ecological factors, including social environments, cognitive functions, and emotional states, have significantly impacted decision-making abilities. However, existing literature primarily reflects Western contexts, neglecting diverse cultural and socioeconomic influences in non-Western settings such as Pakistan.

This study addresses this gap by employing a neurocognitive approach to investigate ecological and cognitive determinants of decision-making among Pakistani older adults. By analyzing the relationships between demographic variables (age, gender, marital status), living situations, and ecological contexts, this research contributes valuable insights into the culturally specific dynamics that influence decision-making competence in older populations. These insights aim to inform targeted interventions and policy formulations that support independent living and enhance the quality of life among older adults.

## Materials and methods

The quantitative cross-sectional study aimed to assess the ecological factors influencing decision-making in older adults through a neurocognitive approach. We designed a structured questionnaire distributed to older adults aged 45 and above in Pakistan. The participants were selected based on their English literacy and ability to complete the questionnaire independently. Therefore, the questionnaire was administered physically, ensuring the respondents' ease of access and convenience.

Participants were recruited in person from clinical facilities in the twin cities of Rawalpindi and Islamabad. The research team directly approached individuals in outpatient departments and community mental health centers, identifying potential participants based on the predefined inclusion criteria: adults aged 45 years and above who were literate in English and able to independently complete the questionnaire. After confirming eligibility, the purpose and procedures of the study were explained, and written informed consent was obtained. Recruitment was conducted between September and October 2024. One hundred twenty adults participated, of whom 100 met the inclusion criteria and were included in the final analysis. This sample was subdivided based on age groups as follows: middle-aged adults, 45-54 years; pre-seniors, 55-64 years; seniors/elderly, 65+ years.

Measures

Measures include a demographic sheet to collect the background information of participants. It includes age, gender, the highest level of education, living situation, current employment, marital status, health, and cognitive status. The first-hand primary source data questionnaire included several validated scales to measure various constructs related to decision-making and well-being. These scales comprised of the following: Adult Decision-Making Competence scale (A-DMC); the A-DMC consists of six validated components including Resistance to Framing (RC1, A1, RC2, A2), Recognizing Social Norms, Under/Overconfidence (CAL), Applying Decision Rules (DR), Consistency in Risk Perception (RP) and Resistance to Sunk Costs (SC). Only RC1 and RC2 questions relevant to our study were incorporated into the questionnaire. The ADMC was designed to assess how well individuals make decisions. It is a close relative of the Youth Decision Making Competence (YDMC) measure developed by Parker and Fischhoff (2005). It was developed with the Decision Outcome Inventory (DOI) (Bruine de Bruin et al., 2007), which assesses whether individuals reach satisfactory outcomes [20]. 

Satisfaction with the Life Scale (SWLS), shown in Appendix 1. The SWLS was developed by Diener et al. (1985) to assess Satisfaction with people's lives. It consists of five statements with a seven-point scale. [21]. The Geriatric Depression Scale (GDS), shown in Appendix 2, created by Yesavage et al., is a brief, 30-item questionnaire in which participants are asked to answer yes or no regarding how they felt over the past week. A short-form GDS consisting of 15 questions was developed in 1986. The GDS was found to have a 92% sensitivity and an 89% specificity when evaluated against diagnostic criteria. Comparing the Long and Short Forms of the GDS for self-rating of symptoms of depression, both were successful in differentiating depressed from non-depressed adults with a high correlation (r = 0.84, p < 0.001) (Sheikh and Yesavage, 1986). We utilized the short form [22]. Additionally, we incorporated items related to decision-making in daily activities, health and well-being, ecological momentary assessment, and cognitive momentary assessment.

Inclusion and exclusion criteria

Adults aged 45 years and older who were literate and able to communicate in English were included in the study; individuals younger than 45 years or those who were illiterate were excluded.

Statistical analysis

All statistical analyses were conducted using SPSS Statistics (v. 26, IBM Corp., Armonk, NY). Categorical variables were analyzed using chi-square (χ²) tests to assess associations between demographic characteristics (e.g., gender, education, marital status, employment status, and age) and categorical outcomes such as context, reason, and accompaniment. Continuous variables were examined using independent samples t-tests (for two-group comparisons, e.g., gender and employment status) and one-way ANOVAs (for comparisons across three or more groups, e.g., age groups, living status, and nature of decision-making). Effect sizes were calculated using Cohen’s d for t-tests and eta squared (η²) for ANOVAs to interpret the magnitude of differences. Pearson’s correlation coefficients were used to assess intercorrelations among continuous psychological, cognitive, and environmental variables. A significance threshold of p < 0.05 was applied for all statistical tests. Missing data were minimal and handled using pairwise deletion to preserve available information across analyses.

Ethical considerations

This study was approved by the ethical research committee of the Brain Tech Clinic and Research Center, prior to sample collection, with the reference no. 2024-2181. The research was conducted according to the guidelines of good clinical practice. Data was handled confidentially and anonymously; individual identifiers were excluded from the collection tool.

## Results

Table 1 presents the distribution and associations of demographic variables (gender, education, marital status, employment status, and age) with the contexts, reasons, and accompanying individuals reported by respondents. There were no significant differences by gender regarding the context, reasons, or accompanying individuals (all p-values > 0.05). Both males and females predominantly reported home as the context, social reasons as the primary reason, and family as the most common accompaniment. Education level did not show significant associations with any of the variables (context, reason, or accompaniment), although respondents without formal education reported slightly more varied contexts. Marital status showed significant associations with the reasons for the experiences (p = 0.001) and accompanying individuals (p = 0.004). Married individuals more frequently cited financial reasons and were more often accompanied by family, while single individuals more often mentioned social reasons and demonstrated more variability in companionship. Employment status did not show significant associations with context, reasons, or accompaniment (all p-values > 0.05). Both employed and unemployed individuals predominantly reported home as the setting, social reasons for their experiences, and were commonly accompanied by family. Age showed a significant association only with accompanying individuals (p = 0.041). Older adults were more frequently accompanied by family than pre-seniors or elderly individuals. There were no significant differences in context or reasons across age groups. In summary, marital status and age emerged as significant variables influencing reasons and companionship, while gender, education, and employment status did not demonstrate statistically significant differences.

**Table 1:** Descriptive statistics of demographic variables f=frequency; %=percentage; p=significance x²=chi square, Chi-square (χ²) tests were used to assess associations between categorical variables. Significance was set at p < 0.05, **=p<0.01. *=p<0.05.

Variable	f (%)	Home	Outdoor	Workplace	Public Place	Other	p	χ^2^	Financial	Health-related	Social	Other	p	χ^2^	Alone	Family	Friends	P	χ^2^
Gender	-	76	4	13	4	3	-	-	20	9	17	54	-	-	3	92	5	-	-
Male	56	42	4	7	2	1	0.424	3.868	13	5	10	28	0.779	1.090	1	50	5	0.097	4.654
Female	44	34	0	6	2	2			7	4	7	26			2	42	0		
Education	-	76	4	13	4	3	-	-	20	9	17	54	-	-	3	92	5	-	-
No formal education	12	7	0	2	1	2	0.191	15.997	5	1	2	4	0.585	7.5	1	11	0	0.457	5.706
High school	19	17	0	2	0	0			4	1	5	9			0	17	2		
Bachelor’s	48	35	2	7	3	1			7	4	7	30			2	43	3		
Others	21	17	2	2	0	0			4	3	3	11			0	21	0		
Marital status	-	76	4	13	4	3	--	-	20	9	17	54	-	-	3	92	5	-	-
Single	22	16	0	2	2	2	0.146	6.815	0	1	9	12	0.001**	15.749	3	18	1	0.004*	10.966
Married	78	60	4	11	2	1			20	8	8	42			0	74	4		
Employment status	-	76	4	13	4	3	-	-	20	9	17	54	-	-	3	92	5	-	-
Employed	49	34	2	9	3	1	0.398	4.060	12	7	9	21	0.099	6.266	0	45	4	0.090	4.805
Unemployed	51	42	2	4	1	2			8	2	8	33			3	47	1		
Age	-	76	4	13	4	3	-	-	20	9	17	54	-	-	3	92	5	-	-
Middle-aged adults	46	36	1	7	2	0	0.464	7.698	7	6	9	24	0.374	6.456	1	43	2	0.041*	9.973
Pre-Seniors	42	29	3	5	2	3			12	3	6	21			0	39	3		
Seniors/Elderly	12	11	0	1	0	0			1	0	2	9			2	10	0		

Table 2 illustrates the intercorrelations among various study variables, identifying significant associations relevant to adult decision-making, subjective well-being, and geriatric depression and their relationships with environmental factors, stress, daily activities, health, ecological contexts, and cognitive engagement. Adult decision-making showed strong associations, particularly with geriatric depression, social environment, and daily activity, suggesting these factors substantially influence adults' decision-making abilities. Subjective well-being was significantly associated with social environment, stress, health, and cognitive engagement. It indicates that individuals with better subjective well-being are more positively influenced by their social contexts, experience less stress, have better overall health, and engage more effectively cognitively. Geriatric depression displayed significant relationships with social and physical environments, stress, and overall health and well-being, implying that depressive symptoms in older adults are strongly impacted by their surrounding social and physical contexts, stress levels, and general health status. The social environment was notably connected with stress and health outcomes, highlighting its influence on stress levels and overall well-being. The physical environment also significantly correlated with stress, emphasizing how physical context may contribute to stress experiences. Stress levels exhibited substantial relationships with daily activities, health outcomes, and cognitive engagement, suggesting that heightened stress negatively influences these domains, potentially impacting daily functioning and mental agility. Daily activities significantly influence overall health and well-being, highlighting the importance of engaging in regular activities for maintaining health among adults. Health and well-being are strongly correlated with cognitive engagement, underscoring the close connection between physical health and cognitive functioning. Ecological context did not show significant correlations with other variables, indicating it may independently influence these outcomes, or its impact is minimal compared to other examined factors. Overall, the findings from Table 2 underscore the complex interplay between psychological factors, environmental contexts, daily activities, and cognitive functions in shaping adult decision-making and well-being.

**Table 2:** Intercorrelation between study variables Pearson’s correlation coefficients were calculated. Asterisks denote statistical significance *=p<0.05, **=p<0.01 considered significant

Variable	Adult’s decision making	Subjective well-being	Geriatrics depression	Social environment	Physical environment	Stress	Daily activity	Health and well-being	Ecological context	Cognitive engagements
Adult’s decision making	-	-	-	-	-	-	-	-	-	-
Subjective well-being	0.524	-	-	-	-	-	-	-	-	-
Geriatrics depression	0.994	0.014	-	-	-	-	-	-	-	-
Social environment	0.951	0.006**	0.0**	-	-	-	-	-	-	-
Physical environment	0.307	0.833	0.01*	0.09	-	-	-	-	-	-
Stress	0.38	0.01**	0.01**	0.017*	0.016*	-	-	-	-	-
Daily activity	0.02*	0.896	0.447	0.513	0.986	0.01**	-	-	-	-
Health and well-being	0.823	0.01**	0.025*	0.002**	0.772	0.004**	0.046*	-	-	-
Ecological context	0.669	0.155	0.073	0.825	0.521	0.67	0.715	0.438	-	-
Cognitive engagements	0.387	0.01**	0.046	0.126	0.78	0.001**	0.21	0.01**	0.658	-

Table 3 compares study variables across demographic categories, i.e., gender. In the gender comparison, subjective well-being and social environment showed statistically significant differences between males and females, with males reporting higher subjective well-being and a better perception of social environment than females. Decision-making, depression, physical environment, stress, ecological context, and cognitive engagement showed no significant differences by gender.

**Table 3:** Comparison of study variables with gender SD=Standard Deviation, t=t-value from the independent samples t-test. P=level of significance, 95%CI= 95% Confidence Interval, LL= Lower Limit and UL= Upper Limit, independent samples t-tests were used, and significance was set at p < 0.05, **=p<0.01, **=p<0.05.

Variable	Male	Female	T	P	Cohen d	95%CI	
	Mean(±SD)	Mean(±SD)				LL	UL
Decision-making	44.21± 14.50	42.45 ± 15.45	0.58	0.5	0.12	-4.2	7.72
Subjective well-being	20.21 ± 4.45	17.95 ± 5.13	2.35	0.02*	0.47	0.35	4.16
Depression	6.60 ± 1.94	6.56 ± 1.75	0.1	0.91	0.02	-0.7	0.78
Social environment	5.30± 1.27	4.72 ± 1.45	2.1	0.03*	0.42	0.03	1.11
Physical environment	10.8± 1.05	11.02 ± 2.04	-0.64	0.52	-0.13	-0.83	0.42
Stress	2.80 ± 1.67	2.70 ± 1.84	0.28	0.78	0.06	-0.6	0.8
Ecological context	5.94 ± 0.90	5.79 ± 0.70	0.91	0.36	0.18	-0.17	0.47
Cognitive engagement	4.0 ± 0.93	3.75 ± 1.18	1.5	0.13	0.31	-0.09	0.74

Table 4 compares study variables across demographic categories, i.e., employment. When comparing employed versus unemployed individuals, none of the variables showed statistically significant differences, suggesting employment status did not significantly impact decision-making, subjective well-being, depression, social or physical environments, stress, ecological context, or cognitive engagement.

**Table 4:** Comparison of study variables with employment SD=standard deviation, t=t-value from the independent samples t-test; P = level of significance, 95% CI = 95% confidence interval, LL = lower limit, UL = upper limit, independent samples. T-tests were used, and significance was set at p < 0.05.

Variable	Employed	Unemployed	T	P	Cohen d	95%CI	
	Mean(±SD)	Mean(±SD)	-	-	-	LL	UL
Decision-making	44.24 ±15.15	42.67 ±14.72	0.53	0.6	0.11	40	48.49
Subjective well-being	19.06 ±4.97	19.37 ±4.82	-0.32	0.75	-0.06	17.67	20.45
Depression	6.45 ±1.66	6.73 ±2.03	-0.75	0.46	-0.15	5.98	6.91
Social environment	5.31 ±1.26	4.80 ±1.46	1.85	0.07	0.37	4.95	5.66
Physical environment	10.94 ±1.57	10.88 ±1.57	0.19	0.85	0.04	10.5	11.38
Stress	3.00 ±1.80	2.53 ±1.68	1.35	0.18	0.27	2.5	3.5
Ecological context	5.92 ±0.91	5.84 ±0.73	0.46	0.65	0.09	5.66	6.17
Cognitive engagement	3.98 ±1.15	3.88 ±0.97	0.46	0.65	0.09	3.66	4.3

Table 5 compares study variables across demographic categories, i.e., marital status. Marital status comparisons indicated significant differences in social environment, stress, and cognitive engagement. Married individuals reported significantly better social environments, lower stress levels, and higher cognitive engagement than singles. No significant differences were observed in decision-making, subjective well-being, depression, physical environment, and ecological context. Overall, gender and marital status revealed notable distinctions in certain psychological and environmental perceptions, while employment status appeared to have minimal influence on the variables examined.

**Table 5:** Comparison of study variables with marital status SD = standard deviation, t = t-value from the independent samples t-test, P = level of significance, 95%CI = 95% confidence interval, LL = lower limit, UL = upper limit. Independent samples t-tests were used, and significance was set at p < 0.05, **=p<0.01, **=p<0.05.

Variable	Single	Married	T	P	Cohen d	95%CI	
	Mean(±SD)	Mean(±SD)				LL	UL
Decision-making	41.27 ±13.25	44.05 ±15.33	-0.84	0.41	0.19	40.65	47.45
Subjective well-being	18.27 ±4.41	19.49 ±4.99	-1.11	0.28	0.25	18.38	20.59
Depression	7.23 ±2.16	6.41 ±1.73	1.63	0.11	-0.45	6.03	6.79
Social environment	4.41 ±1.59	5.23 ±1.27	-2.23	0.03*	0.61	4.95	5.51
Physical environment	10.86 ±2.17	10.92 ±1.37	-0.13	0.9	0.04	10.62	11.23
Stress	3.55 ±2.13	2.54 ±1.57	2.06	0.05*	-0.59	2.19	2.89
Ecological context	5.91 ±0.61	5.87 ±0.87	0.23	0.82	-0.05	5.68	6.07
Cognitive engagement	3.23 ±1.31	4.13 ±0.89	-3.04	0.01**	0.91	3.93	4.33

Table 6 presents a detailed analysis of ecological and cognitive factors across three distinct age groups: middle-aged adults, pre-seniors, and seniors/elderly. Decision-making displayed some variation among age groups, with seniors/elderly participants scoring notably lower than middle-aged adults and Pre-Seniors, though this difference was not statistically significant. It suggests a trend toward reduced decision-making capabilities in the older group, which might indicate potential cognitive challenges emerging with advanced age. However, further research with a larger sample may be needed for more precise conclusions. Subjective well-being scores were highest among seniors/elderly, slightly lower in middle-aged adults, and lowest among pre-seniors. Despite these observed differences, they were not statistically significant, implying that subjective well-being is relatively consistent across these age categories. Depression scores were lowest among seniors/elderly and highest among pre-Seniors, although these differences also lacked statistical significance. This indicates similar levels of depressive symptoms across the groups. Social and physical environment scores demonstrated slight decreases with advancing age, but again, differences across age groups were not statistically significant. This may suggest relatively stable environmental perceptions across different ages, potentially influenced by consistent living conditions or shared experiences among the groups. Ecological context scores slightly declined with age, though differences were minimal and not statistically significant, indicating that perceptions regarding ecological contexts are uniform across age groups. Cognitive engagement remained remarkably consistent across the age groups, reflecting little to no variation among middle-aged adults, pre-Seniors, and seniors/elderly. This may highlight stable cognitive involvement or activity levels, irrespective of age. Overall, these results suggest that ecological and cognitive factors are relatively stable and are not significantly affected by age differences within this sample. The observed trends, particularly in decision-making and subjective well-being, may warrant further exploration with larger sample sizes or additional measures.

**Table 6:** Ecological and cognitive factor base variance among age group M=mean, SD=standard deviation, η²=effect size. One-way ANOVA was used. The effect size is reported as eta-squared (η²). Significance was set at p < 0.05.

Variable	Middle-aged adults	Pre-seniors	Seniors/elderly	F(2,97)	η²
	Mean(±SD)	Mean(±SD)	Mean(±SD)		
Decision-making	44.35(±13.33)	45.21(±14.05)	33.75(±20.33)	3.05	0.01
Subjective well-being	18.93(±4.77)	18.86(±5.18)	21.58(±3.73)	1.63	0.06
Depression	6.65(±1.75)	6.74(±1.91)	5.83(±1.99)	1.16	0.25
Social environment	5.20(±1.26)	5.00(±1.36)	4.67(±1.87)	0.74	0.28
Physical environment	11.11(±1.67)	10.79(±1.32)	10.58(±1.98)	0.76	0.24
Ecological context	5.96(±0.82)	5.86(±0.84)	5.67(±0.78)	0.62	0.48
Cognitive engagement	3.87(±1.15)	3.98(±0.95)	4.00(±1.13)	0.14	0.11

Table 7 presents a detailed analysis of ecological and cognitive factors based on living status (Alone, Family, Friends). Decision-making scores showed notable variation based on living conditions, with individuals living with friends exhibiting the highest scores, followed by those living with family, and the lowest among those living alone. Although suggestive of meaningful differences in decision-making capability depending on companionship, this variation did not reach statistical significance. Subjective well-being displayed statistically significant variance among living conditions. Individuals living with family reported the highest subjective well-being scores, significantly higher than those living with friends, who reported the lowest scores. Those living alone fell in between, though closer to the friend group, indicating a potential protective influence of family companionship on subjective well-being.

**Table 7:** Ecological and cognitive factor base variance among living status M=mean, SD=standard deviation, η²=effect size, **=p<0.01, one-way ANOVA were used and the effect size is reported as eta-squared (η²). Significance was set at p < 0.05 All variables are M(±SD) unless specified otherwise.

Variable	Alone	Family	Friend	F (2,97)	η²
Decision-making	24.33(±13.05)	43.66(±14.64)	50.80(±13.37)	3.23	0.02
Subjective well-being	17.33(±7.09)	19.61(±4.53)	13.20(±6.69)	4.65	0.17
Depression	9.33(±1.53)	6.49(±1.84)	6.80(±1.10)	3.64	0.53
Social environment	1.67(±1.15)	5.20(±1.25)	4.40(±1.14)	12.39	0.89
Physical environment	9.33(±3.79)	11.02(±1.45)	9.80(±1.30)	3.14	0.57
Ecological context	6.33(±0.58)	5.82(±0.81)	6.80(±0.45)	4.14	0.87
Cognitive engagement	3.67(±1.53)	3.97(±1.05)	3.40(±0.89)	0.78	0.41

Depression scores showed substantial differences, with significantly higher levels among individuals living alone, while those living with family or friends reported relatively lower depressive symptoms. This underscores the psychological benefits associated with living with others. Social and environmental scores varied significantly based on living status. Individuals living with family reported the most favourable social environments, significantly higher than those living with friends or alone, who reported considerably less favourable social conditions. This suggests the vital role of family support in enhancing social perceptions. Physical environment scores also indicated significant differences, with individuals living with family reporting higher satisfaction than those living alone or with friends, who reported lower satisfaction levels. This highlights that living with family could positively influence perceptions of one's physical surroundings. Ecological context scores revealed significant differences, where individuals living with friends reported the highest positive ecological perceptions, followed by those living alone and those with family. This indicates diverse ecological perceptions based on different living arrangements. Cognitive engagement scores showed no significant differences across different living statuses, suggesting stable cognitive engagement levels regardless of living arrangements.

Overall, living arrangements significantly influence subjective well-being, depressive symptoms, social environment perceptions, physical environment perceptions, and ecological contexts, highlighting the importance of companionship type in shaping psychological and environmental experiences.

Table 8 provides a detailed analysis of ecological and cognitive factors based on the nature of decision-making (Financial, Health-related, Social, and Other). Decision-making scores were highest for financial decisions, indicating a higher degree of decision-making capability or clarity than health-related, social, and other categories. However, these differences were not statistically significant. Subjective well-being varied slightly among decision-making categories, with the highest subjective well-being scores associated with health-related decisions. Despite this observed trend, these differences were not statistically significant. Depression scores indicated higher levels of depressive symptoms in health-related and financial decision-making groups and relatively lower levels in the social and other categories. However, differences across these categories were not statistically significant. Social and environmental perceptions appeared most favourable among those making financial decisions and least favourable in the social category, although these variations were statistically non-significant. Physical environment scores were highest among those making decisions categorized as "other," suggesting they may have more positive perceptions of the physical environment. Nonetheless, the differences observed across decision categories were not statistically significant. The ecological context was rated highest among participants making financial decisions, suggesting potentially better ecological perceptions or environmental interactions, although these differences did not reach statistical significance. Cognitive engagement scores showed minor variation, with health-related decision-makers reporting slightly higher engagement than other groups, yet these differences also lacked statistical significance. Overall, the results indicate minor but non-significant variations in ecological and cognitive factors based on the nature of decision-making. Further research with larger sample sizes or additional variables could provide deeper insights into these relationships.

**Table 8:** Ecological and cognitive factor base variance among nature of decision-making M=mean, SD=standard deviation, η²=effect size, **=p<0.01. One-way ANOVA were used; effect size is reported as eta squared (η²). Significance was set at p < 0.05.

Variables	Financial	Health-related	Social	Other	F (2,97)	η²
	Mean(±SD)	Mean(±SD)	Mean(±SD)	Mean(±SD)		
Decision-making	50.00(±10.86)	41.78(±18.45)	41.18(±13.17)	42.00(±15.72)	1.67	0.01
Subjective well-being	17.75(±6.46)	20.67(±3.16)	18.65(±4.08)	19.70(±4.62)	1.13	0.05
Depression	7.10(±2.25)	7.44(±2.46)	6.59(±1.66)	6.26(±1.59)	1.75	0.34
Social environment	5.35(±0.93)	5.11(±1.83)	4.59(±1.33)	5.07(±1.45)	0.96	0.33
Physical environment	10.55(±1.76)	10.44(±1.01)	10.47(±1.23)	11.26(±1.61)	2.03	0.46
Ecological context	6.25(±0.85)	5.78(±0.83)	5.88(±0.60)	5.76(±0.85)	1.84	0.74
Cognitive engagement	4.15(±1.18)	4.44(±0.73)	3.71(±1.16)	3.83(±1.00)	1.42	0.56

**Figure 1:**
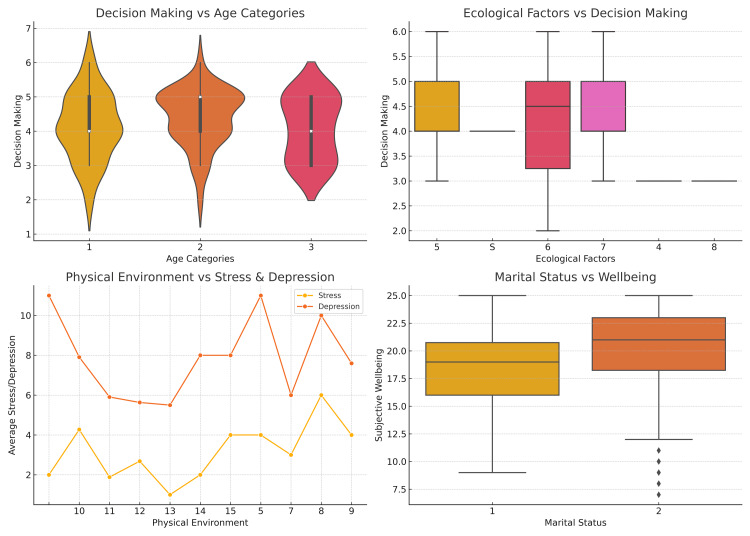
Visualization of decision-making patterns, ecological influences, psychological factors, and subjective well-being about age categories, ecological context, physical environment, and marital status

Figure 1 illustrates differences in decision-making across various age categories. The distributions indicate that decision-making scores appear highest among the middle age group (category 2) with a broader range, suggesting variability in decisional confidence or complexity within this age group. Younger and older adults demonstrate more concentrated and slightly lower decision-making scores, indicating less variability in decision-making patterns or experiences. In the second panel (top right), decision-making across different ecological factors shows significant variability. Certain ecological factors (particularly factors 5, 6, and 7) exhibit greater ranges, indicating that ecological contexts notably influence decision-making processes.

In contrast, factors 4 and 8 show minimal variation, suggesting that these ecological contexts may influence participants' decision-making behaviours less. The third panel (bottom left) explores relationships between the physical environment and psychological indicators of stress and depression. Depression scores generally decrease as physical environment scores improve, suggesting that enhanced physical environments may contribute positively to mental health. Stress scores also fluctuate with the physical environment, though this relationship appears weaker compared to depression, suggesting that depression may be more sensitive to physical environmental factors. Finally, the fourth panel (bottom right) compares subjective well-being between single (category 1) and married (category 2) individuals. Married individuals generally display higher levels of subjective well-being and greater consistency in responses. Single individuals, however, present lower average well-being and more considerable variability in responses, indicating marital status is a potentially important factor in influencing subjective well-being. These visualizations collectively highlight the significance of demographic, ecological, and psychological contexts in influencing decision-making and psychological well-being.

## Discussion

The study aimed to assess the ecological factors influencing middle-aged and older adults' decision-making using a neurocognitive approach. Understanding how aspects of daily life, such as social environment, emotional state, stress, and cognitive status, impact middle-aged and older adults' subjective well-being and decision-making abilities in Pakistan was the primary goal. Using a structured questionnaire that included validated measures like the Adult Decision-Making Competence (A-DMC), Satisfaction with Life Scale (SWLS), and Geriatric Depression Scale (GDS), the study explored the relationship between psychological well-being, decision-making ability, and variables such as stress levels and social environments.

The present study suggested significantly higher subjective well-being in men, which aligns with previous findings showing lower well-being among women [1]. Social pressures may contribute to gender disparities in well-being, as women often manage multiple responsibilities, increasing stress levels. Furthermore, gender differences in coping strategies may explain why men reported higher well-being. However, gender did not show significant differences in the context, reasons, or accompaniment related to decision-making scenarios in the current study.

A significant difference was observed between single and married participants regarding their social environment, stress, and cognitive engagement. These findings are consistent with research reporting better psychological well-being in married individuals compared to singles [23], potentially due to a stronger sense of belonging and support through spousal relationships. Another study similarly reported higher cognitive impairment among single, widowed, and divorced individuals [24], which may be due to reduced emotional, financial, and social support.

This study found descriptively lower decision-making scores in senior participants, which is in line with research indicating that those from this age group perform worse in decision-making, particularly in choice-independent tasks [25]. While our findings did not reveal significant differences in context or reason based on age, middle-aged adults were more frequently accompanied by family, suggesting age-related differences in social behavior and support networks. Age-related cognitive changes, such as declines in working memory and processing speed as well as decreased confidence in decision making, may also contribute to this pattern [26].

The study also identified a significant relationship between participants' social environments and decision-making ability, particularly among those with close friendships. This aligns with previous research showing that extroverts, who are more socially engaged, often demonstrate better decision-making skills due to greater confidence and exposure to diverse viewpoints [27]. Regular social interaction may support collaborative problem-solving, which in turn can enhance decision-making abilities.

Furthermore, the study supports the association between social interaction, subjective well-being, and reduced depression. These findings are consistent with those of Abdullahi et al. [28], who found that participants with open personalities had higher levels of social well-being and overall subjective well-being. Engaging in regular social interaction may provide emotional support, reduce loneliness, and increase resilience. People with more open and socially active personalities may be more likely to pursue stimulating interactions, which supports both psychological and emotional health [26].

In summary, while ecological and social variables were expected to influence decision-making, the results suggest that marital status and age may play more notable roles in shaping how middle-aged and older adults experience their environments and make decisions. These findings underscore the importance of social context and personal relationships in understanding psychological well-being and decision-making in older populations and point to the need for more comprehensive models that incorporate cognitive, emotional, and interpersonal factors.

The study on ecological factors influencing middle-aged and older adults' decision-making has limitations that could compromise the significance and generalizability of the results. First, non-probability purposive sampling may limit the conclusions' generalizability to a broader population. Because the study sample does not fully represent the diversity of older adults, especially in Pakistan, it is challenging to extrapolate results outside of it due to the likelihood of bias. Furthermore, because participants could not remember past events accurately or might alter their responses to perceived social expectations, relying only on self-reported data may lead to self-reporting biases. This issue, commonly occurring in survey-based research, could distort the assessment of the factors influencing judgment. Another limitation of the study is its focus on middle-aged and older adults who are literate and speak English; this leaves out a sizable section of the population and could skew the results by decreasing the sample's representativeness and diversity. Lastly, the cross-sectional methodology of the study only documents a single point in time, which makes it challenging to track changes in ecological factors and decision-making over time or draw conclusions about causal relationships.

## Conclusions

Future research could address these constraints to provide more comprehensive and trustworthy insights. Expanding the sampling technique to incorporate random sampling and a more diverse population would provide a more thorough representation across socioeconomic groups, languages, and origins, making the results more universally relevant. By performing longitudinal studies, researchers can examine causality and the effects of ageing on cognitive and emotional states. These studies may also yield valuable insights into how ecological elements and decision-making evolve. Additionally, objective assessments, such as neurocognitive testing or observational data, may lessen the biases associated with self-reporting and accurately represent the cognitive and emotional factors influencing judgment. Examining how culture influences decision-making can help us better understand ecological factors in older adults in specific contexts, such as Pakistan. Finally, future research may consider other factors, such as technological literacy and support systems, that significantly influence ecological momentary perceptions and decision-making. Addressing these elements may offer a more thorough understanding of decision-making processes and increase the practical implications for healthcare and social support services for older adults.

## Appendices

Appendix 1

The Satisfaction With Life Scale (SWLS) was developed by Ed Diener et al. (1985) to assess satisfaction with people's lives (Table 9).

**Table 9:** Satisfaction with life scale Developed by Ed Diener et al. [21]

Items	1 = Strongly Disagree	2	3	4	5	6	7 = Strongly Agree
In most ways, my life is close to my ideal	-	-	-	-	-	-	-
The conditions of my life are excellent.	-	-	-	-	-	-	-
I am satisfied with my life.	-	-	-	-	-	-	-
So far, I have gotten the important things I want in life.	-	-	-	-	-	-	-
If I could live my life over, I would change almost nothing.	-	-	-	-	-	-	-

Appendix 2

The Geriatric Depression Scale is described in Table 10.

**Table 10:** Geriatric Depression Scale (short form) Developed by Yesavage et al. [22]

Question	Yes	No
Are you satisfied with your life?	-	-
Have you dropped many of your activities and interests?	-	-
Do you feel that your life is empty?	-	-
Do you often get bored?	-	-
Are you in good spirits most of the time?	-	-
Are you afraid that something terrible is going to happen to you?	-	-
Do you feel happy most of the time?	-	-
Do you often feel helpless?	-	-
Do you prefer to stay at home rather than go out and do things?	-	-
Do you feel that you have more problems with memory than most?	-	-
Do you think it is wonderful to be alive now?	-	-
Do you feel worthless the way you are now?	-	-
Do you feel full of energy?	-	-
Do you feel that your situation is hopeless?	-	-
Do you think that most people are better off than you are?	-	-
